# Automatic detection of image sharpening in maxillofacial radiology

**DOI:** 10.1186/s12903-021-01777-9

**Published:** 2021-08-19

**Authors:** Lazar Kats, Yuli Goldman, Adrian Kahn

**Affiliations:** 1 Department of Oral Pathology, Oral Medicine and Maxillofacial Imaging, School of Dental Medicine, Tel Aviv University, 69978 Tel Aviv, Israel; 2 Department of Oral and Maxillofacial Surgery, School of Dental Medicine, Tel Aviv University, Tel Aviv, Israel

**Keywords:** Sharpening, Automatic sharpening detection, Neural network, Maxillofacial radiology, Sharpening detection, Sharpening artifacts

## Abstract

**Background:**

Improvement of image quality in radiology, including the maxillofacial region, is important for diagnosis by enhancing the visual perception of the original image. One of the most used modification methods is sharpening, in which simultaneously with the improvement, due to edge enhancement, several artifacts appear. These might lead to misdiagnosis and, as a consequence, to improper treatment. The purpose of this study was to prove the feasibility and effectiveness of automatic sharpening detection based on neural networks.

**Methods:**

The in-house created dataset contained 4290 X-ray slices from different datasets of cone beam computed tomography images were taken on 2 different devices: Ortophos 3D SL (Sirona Dental Systems GmbH, Bensheim, Germany) and Planmeca ProMax 3D (Planmeca, Helsinki, Finland). The selected slices were modified using the sharpening filter available in the software RadiAnt Dicom Viewer software (Medixant, Poland), version 5.5. The neural network known as "ResNet-50" was used, which has been previously trained on the ImageNet dataset. The input images and their corresponding sharpening maps were used to train the network. For the implementation, Keras with Tensorflow backend was used. The model was trained using NVIDIA GeForce GTX 1080 Ti GPU. Receiver Operating Characteristic (ROC) analysis was performed to calculate the detection accuracy using MedCalc Statistical Software version 14.8.1 (MedCalc Software Ltd, Ostend, Belgium). The study was approved by the Ethical Committee.

**Results:**

For the test, 1200 different images with the filter and without modification were used. An analysis of the detection of three different levels of sharpening (1, 2, 3) showed sensitivity of 53%, 93.33%, 93% and specificity of 72.33%, 84%, 85.33%, respectively with an accuracy of 62.17%, 88.67% and 89% (*p* < 0.0001). The ROC analysis in all tests showed an Area Under Curve (AUC) different from 0.5 (null hypothesis).

**Conclusions:**

This study showed a high performance in automatic sharpening detection of radiological images based on neural network technology. Further investigation of these capabilities, including their application to different types of radiological images, will significantly improve the level of diagnosis and appropriate treatment.

## Background

Digitalization has entered almost every aspect of our lives, including the field of medicine. One of the main areas subject to this process is image processing. Various methods of image processing enable a wide range of modifications, some of which are designed to improve the acquired images. Conversely, the varied capabilities of current image modification techniques make it difficult to determine if changes have been made to the original image.

In dentistry, a large number of direct and indirect digital systems are in use, making image acquisition much easier and quicker [1]. Ultimately, the process of digitalization improves diagnostic possibilities, treatment planning, and as a result, the treatment efficiency. Various images are used in dentistry, including clinical and radiological images. Digital X-rays depend on a large number of settings both in the devices themselves and in the image display and processing software. Built-in and additional programs are used for image processing. Image processing includes a wide variety of algorithms for modifying images, one of which is image enhancement [2].

**Fig. 1:**
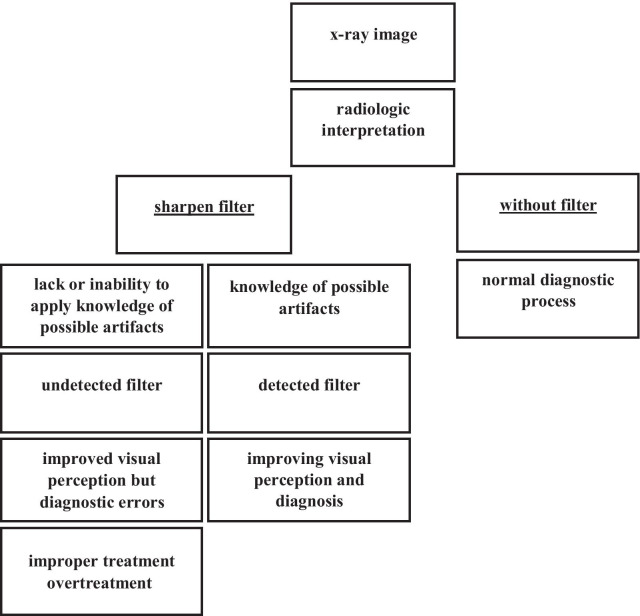
A flow-chart of the possible algorithm of the diagnostic process with the additional influence of the filter application

Image enhancement in radiology, including the maxillofacial region, is of enormous importance in diagnosis, improving the visual perception of the original image. One of the most used modification techniques is sharpening, in which edge enhancement occurs. Unsharp Masking is the basic and most common technique used for sharpening. This method subtracts a smoothed version of the image from the same original image [2]. The result is an improved and sharper image. Certain psychophysical experiments show that edge-enhanced images, including the field of radiology, are often more pleasing to the human visual system [2]. However, the application of this modification algorithm leads to several artifacts [3]. The presence of artifacts in the radiological images can lead to misdiagnosis and, consequently, to improper treatment [4, 5]. The X-ray imaging software used in dentistry includes options to change the initial settings, and if there is insufficient knowledge or experience in interpretation, a misdiagnosis can be made, as shown in the flow-chart of the possible diagnostic process, as presented in Fig. 1.

**Fig. 2:**
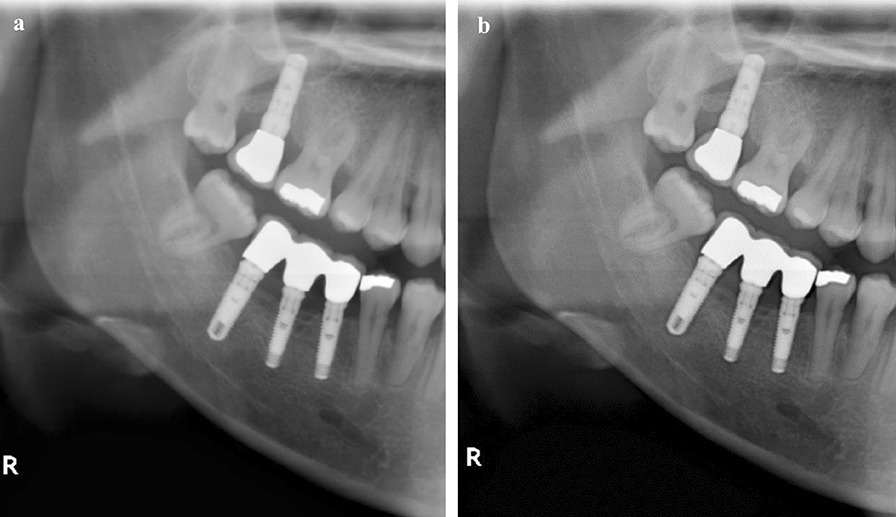
Comparison of the original image **a** with the same image in which the sharpening was applied **b** reveals radiolucent rim formation around fillings, crowns, and implants

The use of sharpening can lead to changes in radiological features of pathological formations, incorrect diagnosis of secondary caries processes, erroneous assessment of osseointegration of dental implants, and some other diagnostic errors (Fig. 2).

The main factor of these changes is an effect called “halo,” “rebound” or “Uberschwinger” artifacts [4], which is a radiolucent rim on the transition border between structures with more and less pronounced densities (Fig. 2). If the difference in densities is more pronounced, the artifact expression is more significant.

In recent years, we can note the important processes of research and application of various algorithms related to the development of technologies and associated with the creation of artificial intelligence. Innovations in this field are based basically on machine learning methods and mainly on deep learning techniques. One of the most important fields of application of these technologies is medicine, and in particular, dentistry, where many applications have been developed based on deep learning in order to solve problems of diagnosis, choice of a treatment program, and other tasks [6]. The main applications of neural networks are classification, detection and segmentation algorithms. The classification algorithm is fundamental and predicts which class a particular image belongs to. Currently, it can be considered that neural networks are the best method of automatic classification of images [7].

Neural networks as a basis for deep learning have shown particular success in radiology [8]. Much attention is being paid to the use of computer-assisted diagnosis for the classification of X-rays, automatic detection and segmentation of various pathologies [8]. In the field of dentistry several studies, ranging from the identification of caries to the detection of cysts and tumors, have also confirmed the effectiveness of the application of neural networks [9].

Various methods have been used to determine sharpening in radiology, including methods based on overshoot artifacts analysis [10], feature-based methods [11], and multiresolution overshoot artifact analysis [12]. Recently, the use of neural networks to detect possible sharpening has quite logically been tested, with very encouraging results [13].

To the best of our knowledge, this kind of peer-review study has not yet been done in the field of maxillofacial radiology with successful automatic identification of the fact that a filter has been applied, which could aid in preventing misdiagnosis and, as a consequence, improper treatment. The goal of this study was to prove the feasibility and effectiveness of automatic sharpening detection based on neural networks.

## Methods and materials

The study (and the use of X-ray image in the study) been independently reviewed and approved by the Institutional Review Board of Tel-Aviv University on 25/04/2020 (N^0^ 0001273-1).

### Study design

The present study is retrospective. The purpose of this study was to verify the possibility and effectiveness of using a neural network to solve the classification problem and to automatically determine the application of the sharpening filter for an X-ray image. The knowledge that this filter has been applied is necessary to prevent errors in the diagnostic process. Frequently, the dentist receives a X-ray image in a certain format and has no way to view the history of applied changes. Our objective was to use deep learning, as a result of which the neural network was trained on the database of images without changes and images with filter application. The input for the finished algorithm was a X-ray image that passes through the trained neural network and the output was a binary classification including the image with or without filter application. Thus, if successful, the resulting prediction model would automatically determine that a filter has been applied to the incoming image. If the predictions result in a positive prediction, the dentist would know that a number of radiologic manifestations should be considered as artifacts, thus avoiding misdiagnosis.

### Data

We used an in-house dataset created at the School of Dental Medicine, Tel Aviv University.

The dataset contained 4290 X-ray slices randomly selected from different datasets of cone-beam computed tomography (CBCT) images performed on two different devices: Ortophos 3D SL (Sirona Dental Systems GmbH, Bensheim, Germany) and Planmeca ProMax 3D (Planmeca, Helsinki, Finland). This dataset was prepared for the purpose of this research and has not been previously used in other studies. None of the slices contained any annotations; personal data were anonymized during dataset preparation. No clinical or demographic information was saved for future use. Two oral medicine specialists with expertise in oral and maxillofacial radiology were involved in the image collection and labeling. Initial data selection was performed from the original datasets without changing any software settings and without applying any filters. The selected slices were modified using the sharpening filter available in the software RadiAnt Dicom Viewer software (Medixant, Poznan, Poland), version 5.5. One of the slices, axial, coronal or sagittal, was used as a single image. The slices were not pre-cropped and were used as complete images. The number of different slices was the same for the corresponding test. The data set varied considerably depending on the size and shape, on the anatomical structures, and the different levels of contrast and brightness of the images obtained in the two different units. To train the neural network, 4290 original images were used, to which the sharpening of three levels of intensity, present in the RadiAnt Dicom Viewer, was applied. In this way, 4290 images without changes and 4290 images with the applied filter of a certain intensity, were used at each stage of training. All images were saved with a Viewer in Joint Photographic Experts Group (JPEG) format. Thus, several trained models were obtained for subsequent testing on images that did not participate in the training. The sharpening levels and thus the resulting models were coded as “SH (sharpening) 1”, “SH_2” and “SH_3”, where “SH_1” was the minimum and “SH_3” the maximum filter level in the software. The unmodified images and the corresponding models were coded as “N” (normal). Labeling was done by the same two oral medicine specialists. No additional annotations other than filter application codes were made.

### Data partitions

Thus, four subsets of slices were obtained, including N and SH, 1–3 sharpening levels, and each contained 4290 images, of which 80% were randomly selected for training and 20% for validation. In addition, 1200 slices were used for the test, including 300 of each identified type.

### Model

#### Neural networks architecture

At present, convolutional neural networks (CNNs) are most commonly used to solve computer vision challenges, including the basic image classification problem [14]. In this study, we used a type of one of the most successful and used for these tasks deep neural network known as Residual Networks-50 ("ResNet-50") [15, 16]. This is a 50-layered, residual deep CNN, composed of 5 stages, each of them includes a convolution block and an identity block. In turn, each convolution block and each identity block have 3 convolution layers.

The most proven transfer learning concept, in which a neural network is based on prior knowledge obtained in another similar task of non-profiled image classification, was chosen for the research [17]. In this study, the neural network model was previously trained on the ImageNet dataset [18]. ImageNet is a dataset of over 15 million labeled images across more than 20,000 categories [18]. The model used in this study was pre-trained on a subset of ImageNet including about 1.3 million images belonging to about 1000 categories. The convolution layers, except the last, are followed by a rectified linear units (ReLU). The final classifier using the sigmoid activation function and the output is a binary classification of the presence or absence of application of the sharpening filter.

### Data preprocessing and augmentation

The input images were resized to 224 × 224 pixels. Each image was into 3 channels, thus the input image form was 224 × 224 × 3. Afterwards, the data were normalized using standardization. Augmentation protocols of Keras ImageDataGenerator were used and included randomly horizontally or vertically flipping, rotating images by a range of 15°, height and width shifting by a range of 0.1 and zoom changing by a factor of 0.5 so that the neural network was trained on more examples to prevent overfitting.

### Training details

The input images and their corresponding sharpening maps were used to train the network in a mini-batch manner, with batch size being is set to 32. In this way, 80% of the data were randomly chosen for training and 20% for validation. Gradient descent computation and updates were carried out by Adam optimizer with a fixed learning rate of 0.001 and binary crossentropy as the loss of function for our binary classification problem. The number of epochs was taken as 100. In this way, protocols for stopping the training when overfitting and choosing the best model, were used. Keras with the Tensorflow backend was used for the implementation. The test dataset contained images from 2 different devices: Orthophos 3D SL (Sirona Dental Systems GmbH, Bensheim, Germany) and Planmeca ProMax 3D (Planmeca, Helsinki, Finland). The model was trained using NVIDIA GeForce GTX 1080 Ti GPU.

### Evaluation

The following statistical metrics were used to evaluate this study: Accuracy, Sensitivity and Specificity, calculated according to the following formulas:

$$Accuracy=\frac{\mathrm{N}tn+\mathrm{N}tp}{\mathrm{N}tn+ \mathrm{N}fn+ \mathrm{N}tp+ \mathrm{N}fp}$$

$$Sensitivity=\frac{\mathrm{N}tp}{ \mathrm{N}fn+ \mathrm{N}tp}$$

$$Specificity=\frac{\mathrm{N}tn}{ \mathrm{N}tn+ \mathrm{N}fp}$$

In this study, the positive and negative cases were calculated according to the applied filter or the unchanged image. Therefore, N*tp* and N*tn* represent correctly detected cases with or without a sharpening filter, respectively, while N*fp* and N*fn* represent incorrect detection.

In addition, a Receiver Operating Characteristic (ROC) analysis was performed to calculate the detection accuracy (null hypothesis: true area = 0.5), confidence interval (CI) 95%. The MedCalc Statistical Software version 14.8.1 (MedCalc Software Ltd, Ostend, Belgium) was used for the statistical analysis.

## Results

One-thousand-two-hundred different images (300 in each test type) with filter and without modification were used for the test, which did not participate in the neural network training. Thus, the following pairs of test datasets were obtained: “SH_1 versus N”, “SH_2 versus N” and “SH_3 versus N”.

**Fig. 3:**
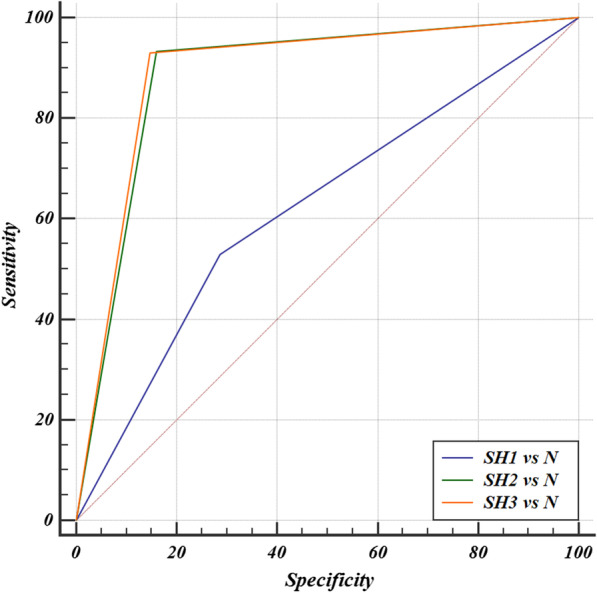
ROC curve, AUC “SH_1 versus N” = 0.622; AUC “SH_2 versus N” = 0.887; AUC “SH_3 versus N” = 0.892. SH: Sharpening; N: Normal

The sensitivity for the detection of sharpening in the pair “SH_1 versus N” was 53% and the specificity was 72.33%, with an accuracy of 62.17% (*p *< 0.0001). The ROC analysis showed an Area Under Curve (AUC) different from 0.5 (null hypothesis) (Table 1, Fig. 3).

**Table 1:** Statistical analysis results

Model	Accuracy (%)	Sensitivity (%)	Specificity (%)	AUC	95% confidence interval	Standard error DeLong et al. 1988	Significance level *p* (area = 0.5)
SH1 versus N	62.17	53	72.33	0.622	0.582 to 0.661	0.0195	< 0.0001
SH2 versus N	88.67	93.33	84.00	0.887	0.859 to 0.911	0.0128	< 0.0001
SH3 versus N	89	93.00	85.33	0.892	0.864 to 0.915	0.0126	< 0.0001

SH: Sharpening; N: Normal

In the second and third pairs, the received values were very similar. The sensitivity, specificity and accuracy in the pair “SH_2 versus N” were 93.33%, 84% and 88.67%, respectively (*p* < 0.0001), while in in the pair “SH_3 versus N” the sensitivity, specificity and accuracy were 93%, 85.33% and 89%, respectively (*p *< 0.0001). The ROC analysis showed a significant AUC different from 0.5 (null hypothesis) (Table 1, Fig. 3).

It is worth noting that in the pairwise comparison of ROC curves of all three pairs of models, there were significant statistic differences between pair “SH_1 versus N” and the others (*p* < 0.0001), while there was no significant difference between the pairs “SH_2 versus N” and “SH_3 versus N” (*p* = 0.7058).

## Discussion

**Fig. 4:**
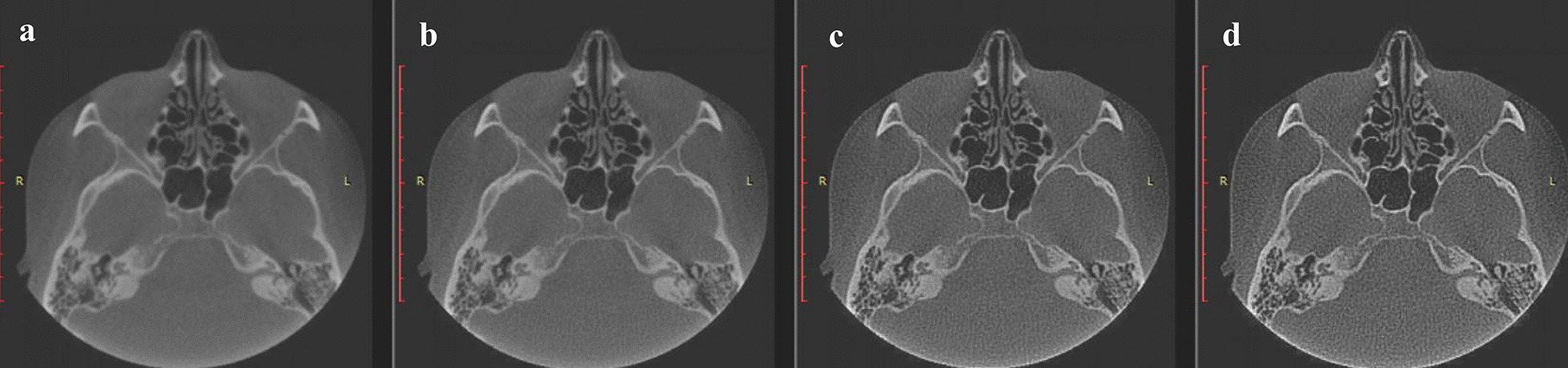
**a** Original image without modification; **b** first level sharpening; **c** second level sharpening; **d** third level sharpening. At the comparison, it is possible to verify the minimal difference of the first (**b**) level of sharpening from the original image (**a**), while the second (**c**) and third (**d**) levels differ significantly from the image without modification

Based on this study, the detection efficiency of applying the sharpening filter has been demonstrated. At the minimum filter level SH_1, the original and modified images were barely distinguishable visually (Fig. 4a, b).

The research results in this variant were the weakest. The sensitivity for the detection of sharpening in pair "SH_1 versus N" was 53% and the specificity was 72.33%, with an accuracy of 62.17% (*p* < 0.0001) (Table 1, Fig. 3). Despite the statistically significant result, it is considerably lower than that of the other sharpening levels and insufficient for practical application. Conversely, this level of difference for both the human visual system and the algorithm was unlikely to produce significant diagnostic artifacts and, as a consequence, was not clinically meaningful.

The main risk associated with failure to detect this filter as diagnostic errors was that it would lead to improper treatment choices. There are several basic errors of this kind in dentistry. At the border of X-ray contrast dental fillers, the application of sharpening causes the formation of a radiolucent rime, which can be mistaken for a secondary carious process and unnecessary treatment [4, 5]. In some cases, this might lead to the erroneous need for endodontic treatment, without any real indication for it. Using this filter in images of different types of prosthetic treatments, including crowns and dental bridges containing metal parts, could lead to the illusion of secondary processes [5, 19]. The treatment tactics in this case can represent an absolute overtreatment of non-existent processes and performing the entire prosthetic cycle again. In the case of metal dental implants, the "halo" formed around them might mistakenly suggest a lack of osseointegration and entail unnecessary treatment, including possible removal of the implants [20]. It is also important to highlight the possible changes that might emerge in the differential diagnosis of bone-related pathological formations as a consequence of changes in their radiological features [20]. Any of these consequences might result in jeopardizing patients' health, lowering the level of medical care and raising economic and social impacts, for both patients undergoing unnecessary treatments and medical staff.

In the case of SH_1, the occurrence of the artifact is unlikely and weakly pronounced. In the stronger SH_2 and SH_3 filters, the changes are pronounced to a large extent (Fig. 4c, d).

It should be noted that the results at these levels were very similar: the sensitivity, specificity and accuracy in the pair “SH_2 versus N” were 93.33%, 84% and 88.67%, respectively (*p* < 0.0001), while in in the pair “SH_3 versus N” the sensitivity, specificity and accuracy were 93%, 85.33% and 89%, respectively (*p *< 0.0001); the ROC analysis showed a significant AUC different from 0.5 (null hypothesis) (Table 1, Fig. 3). It is important to emphasize that this study tested existing sharpening levels without the possibility of fine-tuning. Most of the current dicom viewers have built-in capabilities to apply and modify filters without the possibility of fine-tuning of the basic characteristics of the filter [3]. In our study, special importance was given to clinical use and not just to theoretical testing of automatic detection capability. That is why one of the routine dicom viewers was chosen, rather than a specialized image-processing program with full user control of the settings. Thus, different viewers have filters with different initial settings without standardization. Using another program, the possibilities and levels of sharpening are different and therefore the study did not have versatility to the large variety of existing programs. In addition, it is also important to note that there are many manufacturers of a variety of X-ray units, but only two were used in this study. As a result of the lack of standardization, the initial settings in the hardware and software could differ greatly, a fact that affects the initial original images and their possible modifications.

Research on automatic sharpening detection has been conducted for more than a decade, along which several effective ways have been proposed, however the number of papers on this issue is still very limited [13]. Currently, the most successful and effective method is the use of neural networks, which have shown their competence, notwithstanding the rare specialized studies on the topic of automatic sharpening detection [13]. The investigation that showed the best results used a non-medical database of non-compressed grayscale images and achieved a maximum accuracy of over 99% using the original neural network structure proposed by the authors [13]. In our study, the maximum accuracy was 89%. A possible explanation for this discrepancy could be differences in the architecture of the neural network and training technology. However, the main point of difference seems to be the use of ready levels of a built-in filter and the inability to fine-tune the properties of the filter. As already mentioned, this could be considered as an advantage, because it is the only existing possibility for viewers used in practice. In this work, one of the most successful neural network architectures, "ResNet-50", has been used, which has proven its effectiveness for image classification tasks, including Image forensics, one of whose tasks is the detection of graphical modifications made to the original image [15, 16, 21]. An additional important aspect to mention is the application of the transfer deep learning used in this study, in which the neural networks did not start training from scratch, but were pretrained on other images, which significantly improved the final result of automatic classification [22].

Unfortunately, there is a limited research in this field, with no peer-reviewed studies in dentistry, thus precuding the possibility to perform a comparative characterization with similar studies. Dental practices are most often private and therefore, dentists need to make diagnostic and treatment decisions on their own, usually without chair-side possibility to consult with colleagues, if necessary. Therefore, the possibility of computer-assisted automatic detection can provide an alternative way of artificial “second opinion” to improve quality of dental practice.

## Conclusion

Many digital imaging systems are currently used in dentistry for both 2D and 3D images. Software for analyzing and processing the acquired images uses a variety of algorithms to improve the quality of the image, one of which is sharpening. A significant improvement in visual quality is achieved with the filter of sharpening, but at the same time appearance of artifacts is probably inevitable. If dentists are unaware of the use of this filter and there is a lack of experience in the interpretation of the emerging artifacts, misdiagnosis is possible and, as a consequence, improper treatment could follow. The incredibly rapid development of computer vision tools, based on deep learning neural networks, has made it able to achieve the best results in image identification and classification. This study shows most positive results in automatic sharpening detection based on neural networks. Further investigation of these capabilities, including their application to different image modalities obtained with different units, will significantly improve the level of diagnosis and appropriate treatment.

## Data Availability

The datasets used and/or analyzed during the current study are available from the corresponding author on reasonable request.

## Abbreviations

ROC analysis: Receiver operating characteristic analysis
AUC: Area under curve
CNN's: Convolutional neural networks
CBCT: Cone beam computed tomography
SH: Sharpening
N: Normal
